# Draft genome of an *Escherichia coli* gut isolate from a sertraline-treated patient suggests potential antibiotic resistance induction

**DOI:** 10.1128/mra.01087-25

**Published:** 2026-03-10

**Authors:** Johanna L. Wallnisch, Tia Wünschmann, Anna Baborski, Moritz Rau, Alexander Refisch, Nils Opel, Rosalind J. Allen, Anne Busch

**Affiliations:** 1 Theoretical Microbial Ecology, Friedrich Schiller University Jena 9378, Jena, Germany; 2 Cluster of Excellence Balance of the Microverse, Friedrich Schiller University Jena 9378, Jena, Germany; 3 Department of Psychiatry and Psychotherapy, University Hospital Jena 39065, Jena, Germany; 4 German Center for Mental Health (DZPG) 675615, Mannheim, Germany; 5 Department of Psychiatry and Neuroscience, Campus Benjamin Franklin, Charité–Universitätsmedizin Berlin 14903, Berlin, Germany; 6 Department of Neurology, University Hospital Jena 39065, Jena, Germany; Nanchang University, Nanchang, Jiangxi, China

**Keywords:** antidepressants, antibiotic resistance, *Escherichia coli*

## Abstract

The draft genome of *Escherichia coli* ADAR08_002, from feces of a patient with major depressive disorder (MDD) treated long-term with sertraline, is 5,247,898 bp (GC 50.65%) in 273 contigs (N50 153,410 bp). It harbors mobile elements and antibiotic resistance, persistence, and metabolic adaptation genes, serving as a resource to study antidepressant impacts.

## ANNOUNCEMENT

We describe *Escherichia coli* ADAR08_002, isolated in Thuringia, Germany, from the feces of a female patient with severe depression (Beck Depression Inventory [BDI] score 28, body mass index [BMI] 22) who had been undergoing continuous treatment with the antidepressant sertraline for over 1 year. Previous work suggests that antidepressant exposure can accelerate the evolution of antibiotic resistance and stress responses (1, 2). Consistent with this concept, the strain exhibits partial phenotypic and genotypic resistance to multiple antibiotics and carries a mobile genetic element.

The organism *E. coli* ADAR08_002 was selected and isolated (MindBodyBrain cohort, AZ:2022-2701-BO) and stored in thioglycolate medium at –70°C until use. The sample was cultured on MacConkey agar (Carl Roth, Karlsruhe, Germany) overnight at 37°C under aerobic conditions. To assess resistance, colonies were plated on MacConkey agar containing European Committee on Antimicrobial Susceptibility Testing (EUCAST)-defined MICs of meropenem, piperacillin/tazobactam, colistin, tigecycline, tobramycin, ciprofloxacin, and ampicillin (3).

The strain was cultured in brain heart infusion (BHI) medium for 24 h at 37 °C prior to DNA extraction. Genomic DNA was isolated using the PureLink Genomic DNA Mini Kit (Invitrogen) (4). Species identity was confirmed by 16S rRNA sequencing (EUROFINS, Ebersberg, Germany) as described before (5, 6). Sequences were compared to reference genomes using NCBI BLASTn, assigning strain identity to *E. coli* with the closest match to *E. coli* O157:H7 str. Sakai (BA000007.2) and tested for contamination EZBio ContEST 16S (7–9). Libraries were prepared using the Illumina DNA Prep Kit with Nextera CD indexes, and paired-end sequencing (2 × 150 bp) was performed on the iSeq platform (10, 11). FastQC was used for read quality assessment (v.0.74) (12). *De novo* assembly was performed using SPAdes v3.15.5, and contigs shorter than 200 bp were removed (13). The genome assembly was assessed with QUAST v5.3.0 (14), and annotation was performed using Prokka v1.14.6 (15). For ADAR08_002, paired-end sequencing yielded 693,138 reads. The genome assembly contained 273 contigs prior to filtering, with an N50 of 153,410 bp. The total genome length was 5,247,898 bp, with a GC content of 50.65%. Reads were mapped to the assembled genome using BWA-MEM2 v2.2.1, and coverage was determined with bamCoverage v3.5.4, explicitly stating that the assembled genome served as reference (16, 17). The draft genome (38× coverage; 99% completeness (estimated with Quast v5.3.0 [14]) consisted of 273 contigs totaling 5,247,898 bp and encoded 4,828 predicted CDSs, five rRNAs, 78 tRNAs, one tmRNA, and two repeat regions. A total of 2,710 reads were associated with the mobile genetic element, of which 2,698 (99.6%) successfully mapped to the reference sequence using the ‘Map to Reference’ function in Geneious Prime v2024.0.7 (18). Unless otherwise specified, all software was used with default parameters.

We identified resistance genes for amoxicillin, ampicillin, piperacillin, ticarcillin, and cephalothin with 99.88% identity via RESfinder (v.4.7.2), suggesting functional resistance potential (19). Additional genes conferring resistance to streptomycin, sulfamethoxazole, trimethoprim, tetracycline, and doxycycline were found with 100% identity via RESfinder (v.4.7.2). IS1397, an indicator of genomic flexibility, was identified by ISfinder (v.2025-06-06) in contigs 116 (complete; coverage 191) and 184 (partial; coverage 112), corresponding to six and four copies, respectively; contig 184 encodes a DDE-type integrase/transposase/recombinase (EOD0051004.1).

## Data Availability

The raw reads and the assembled genomes for the *Escherichia coli* isolate are available for the project under reference PRJNA1285810, Biosample ID SAMN49775784, and SRA SRX29600990 and SRX29600991. This Whole Genome Shotgun project has been deposited at DDBJ/ENA/GenBank under accession JBREGL000000000.1.

## References

[1] Ding P, Lu J, Wang Y, Schembri MA, Guo J. 2022. Antidepressants promote the spread of antibiotic resistance via horizontally conjugative gene transfer. Environ Microbiol 24:5261–5276. doi:10.1111/1462-2920.16165 36054646 PMC9804927

[2] Wang Y, Yu Z, Ding P, Lu J, Mao L, Ngiam L, Yuan Z, Engelstädter J, Schembri MA, Guo J. 2023. Antidepressants can induce mutation and enhance persistence toward multiple antibiotics. Proc Natl Acad Sci USA 120:e2208344120. doi:10.1073/pnas.2208344120 36689653 PMC9945972

[3] European Committee on Antimicrobial Susceptibility Testing. 2024. EUCAST, clinical breakpoints - bacteria. Available from: https://www.eucast.org/fileadmin/eucast/pdf/breakpoints/v_16.0_Breakpoint_Tables.pdf

[4] Invitrogen. 2007. Pure LinkTM genomic DNA mini kit of invitrogen protocol

[5] Dumann G, Rohland O, Abdel-Glil MY, Allen RJ, Bauer M, Busch A. 2024. Draft genomes of the bile duct microbiome strains Klebsiella pneumoniae and Enterococcus lactis isolated from bilioenteric drainages with biofilm-forming abilities. Microbiol Resour Announc 13:e0020224. doi:10.1128/mra.00202-24 39470240 PMC11636323

[6] Baborski A, Rohland O, Wuenschmann T, Bauer M, Allen RJ, Busch A. 2026. Biofilm-derived bile duct microbiota in liver transplantation: high-quality genomes of Klebsiella pneumoniae, Enterococcus faecalis, and Enterococcus faecium Microbiol Resour Announc 15:e0088625. doi:10.1128/mra.00886-25 41568955 PMC12896312

[7] Altschul SF, Gish W, Miller W, Myers EW, Lipman DJ. 1990. Basic local alignment search tool. J Mol Biol 215:403–410. doi:10.1016/S0022-2836(05)80360-2 2231712

[8] Altschul SF, Madden TL, Schäffer AA, Zhang J, Zhang Z, Miller W, Lipman DJ. 1997. Gapped BLAST and PSI-BLAST: a new generation of protein database search programs. Nucleic Acids Res 25:3389–3402. doi:10.1093/nar/25.17.3389 9254694 PMC146917

[9] Lee I, Chalita M, Ha S-M, Na S-I, Yoon S-H, Chun J. 2017. ContEst16S: an algorithm that identifies contaminated prokaryotic genomes using 16S RNA gene sequences. Int J Syst Evol Microbiol 67:2053–2057. doi:10.1099/ijsem.0.001872 28639931

[10] Invitrogen. 2025. Nextera DNA flex microbial colony extraction protocol

[11] Invitrogen. 2025. Nextera DNA flex lLibrary Prep on the iSeq 100 system

[12] Simon Andrews FK, Segonds-Pichon A, Biggins L, Krueger C, Wingett S. 2010. FastQC: a quality control tool for high throughput sequence data. Babraham Institute. http://www.bioinformatics.babraham.ac.uk/projects/fastqc.

[13] Bankevich A, Nurk S, Antipov D, Gurevich AA, Dvorkin M, Kulikov AS, Lesin VM, Nikolenko SI, Pham S, Prjibelski AD, Pyshkin AV, Sirotkin AV, Vyahhi N, Tesler G, Alekseyev MA, Pevzner PA. 2012. SPAdes: a new genome assembly algorithm and its applications to single-cell sequencing. J Comput Biol 19:455–477. doi:10.1089/cmb.2012.0021 22506599 PMC3342519

[14] Gurevich A, Saveliev V, Vyahhi N, Tesler G. 2013. QUAST: quality assessment tool for genome assemblies. Bioinformatics 29:1072–1075. doi:10.1093/bioinformatics/btt086 23422339 PMC3624806

[15] Seemann T. 2014. Prokka: rapid prokaryotic genome annotation. Bioinformatics 30:2068–2069. doi:10.1093/bioinformatics/btu153 24642063

[16] Vasimuddin Md, Misra S, Li H, Aluru S. 2019. Efficient architecture-aware acceleration of BWA-MEM for multicore systems. 2019 IEEE International Parallel and Distributed Processing Symposium (IPDPS); Rio de Janeiro, Brazil. doi:10.1109/IPDPS.2019.00041

[17] Ramírez F, Dündar F, Diehl S, Grüning BA, Manke T. 2014. deepTools: a flexible platform for exploring deep-sequencing data. Nucleic Acids Res 42:W187–91. doi:10.1093/nar/gku365 24799436 PMC4086134

[18] Biomatters Ltd. 2024. Geneious Prime (Version 2024.0.7). Available from: https://www.geneious.com

[19] Bortolaia V, Kaas RS, Ruppe E, Roberts MC, Schwarz S, Cattoir V, Philippon A, Allesoe RL, Rebelo AR, Florensa AF, et al.. 2020. ResFinder 4.0 for predictions of phenotypes from genotypes. J Antimicrob Chemother 75:3491–3500. doi:10.1093/jac/dkaa345 32780112 PMC7662176

